# Group cohesion, motor self-efficacy, and socio-emotional skills in physical education students in a region of Spain: a descriptive study

**DOI:** 10.3389/fpsyg.2025.1631231

**Published:** 2025-07-23

**Authors:** María José García-Guillén, Antonio Castillo-Paredes, Carmen Galán-Arroyo, Jorge Rojo-Ramos

**Affiliations:** ^1^Promoting a Healthy Society Research Group (PHeSO), Faculty of Sport Sciences, University of Extremadura, Cáceres, Spain; ^2^Grupo AFySE, Investigación en Actividad Física y Salud Escolar, Escuela de Pedagogía en Educación Física, Facultad de Educación, Universidad de Las Américas, Santiago, Chile; ^3^BioErgon Research Group, Faculty of Sport Sciences, University of Extremadura, Cáceres, Spain

**Keywords:** physical activity, group cohesion, motor self-efficacy, frustration tolerance, social skills

## Abstract

**Introduction:**

Understanding the impact of physical activity, mobile device use, and sociodemographic variables on students' psychosocial development is key to promoting healthier and more inclusive educational environments. This study examined how factors such as gender, age, geographic location, PA, and mobile device use influence group cohesion, motor self-efficacy, frustration tolerance, and social skills in primary and secondary school students.

**Methods:**

A cross-sectional design was used with a large sample of school-aged children and adolescents. The study assessed group cohesion, motor self-efficacy, frustration tolerance, and social skills through validated scales. Statistical analyses included *t*-tests, effect sizes (Hedges' g), Spearman correlations, and multiple linear regressions to examine relationships and predictive models across sociodemographic and behavioral variables.

**Results:**

Girls scored significantly higher in assertiveness, frustration tolerance, and communication and conflict resolution skills. Rural students outperformed their urban peers across several psychosocial dimensions. Higher PA levels were associated with increased motor self-efficacy and group cohesion, particularly in boys. Excessive mobile device use correlated negatively with communication and conflict resolution skills, especially in girls. Regression analyses revealed that variables such as PA, sex, and mobile device use significantly predicted group cohesion and communication skills. Frustration tolerance and conflict resolution were notably influenced by communication skills and assertiveness, respectively.

**Conclusion:**

Regular engagement in physical activity and reduced mobile device use are positively associated with students' psychosocial competencies. These findings highlight the need for targeted educational interventions that consider students' age, gender, and behavioral habits.

## 1 Introduction

The subject of Physical Education (PE) plays a crucial role in promoting physical activity (PA) in the educational environment by providing an appropriate place to implement strategies that stimulate active behaviors among students (Tomás et al., 2014). Several studies show that the regular practice of physical exercise generates advantages at physical, psychological and social levels (Fabio and Towey, 2018; Mazereel et al., 2021; Mygind et al., 2019; Tikac et al., 2022). In this context, the subject of PE in the educational environment emerges as an essential tool to contribute to compliance with international PA recommendations, in particular those dictated by the World Health Organization (OMS, 2020), which propose at least 60 min per day of moderate to intense intensity PA, for children and adolescents aged 5–17 years, including at least 3 days per week of high-intensity activities. This inactivity has been associated with several detrimental consequences, such as changes in body composition, the onset of harmful behaviors (such as drug use), disinterest in PA, and a lack of social support for staying physically active (Guthold et al., 2020). Faced with this situation, it is essential to adopt a broad vision that includes the interrelation of biological, emotional, social and cognitive elements, which directly affect motivation toward PA and persistence in sport (Moreno Murcia et al., 2008; Mosqueda et al., 2022).

PE not only focuses on the attainment of motor skills and physical growth, but also provides an environment conducive to promoting social interaction and collaborative work, essential components for the development of social skills (Eys et al., 2015). Group cohesion can be defined as an active process in which the members of a group foster a feeling of unity and belonging, aimed at both the realization of shared goals and the satisfaction of the emotional and social needs of its members (Gajardo et al., 2024). This environment of unity and collaboration promotes, in turn, the strengthening of social competencies, defined as the set of interpersonal behaviors and tactics that enable people to interact efficiently, solve conflicts, communicate assertively and build positive relationships with others (Oliva Delgado et al., 2011). PE, by including group activities, becomes a good tool to strengthen cohesion, which can increase motivation and academic performance (Berk, 2018).

In this same framework of integral growth, motor self-efficacy plays a crucial role, since it refers to the perception that the individual has of his or her ability to perform physical or sports activities in the best possible way (Bandura, 1997). Research carried out in this area indicates that perceived motor self-efficacy directly influences the type of physical-sport activity in which students participate and can have an impact on the time and intensity with which they engage in such activity (Hernández Álvarez et al., 2008, 2011). As Hernández Álvarez et al. (2008, 2011) point out, the perception of motor efficacy plays a crucial role in students' behavior in relation to their involvement in PA. Balaguer Solá et al. (1995) point out that those who consider themselves more capable tend to be more motivated to practice sports.

Likewise, another crucial element in the personal growth of students within the context of PE is frustration tolerance, defined as the ability to adaptively manage negative emotions that emerge in adverse circumstances or failure (Ellis, 2006). Individuals with higher frustration tolerance tend to evidence better emotional management and self-worth, in addition to a willingness and ability to adjust to anxious or stressful circumstances (Ibañez et al., 2018). This consequently simplifies organization toward specific goals, problem solving and correct decision making (Ibañez et al., 2018). When frustration is not adequately managed, it can adversely impact social growth, as evidenced by the research of Varela and Mustaca (2021), who found that adults with lower frustration intolerance tend to have better social skills. A similar phenomenon occurs in adolescents, as pointed out by Zavala and Pérez (2022), who emphasize that adolescence is a transitional phase where individuals encounter different stressful circumstances. These experiences can impact how they employ coping tactics and their ability to manage frustration (Varela and Mustaca, 2021; Zavala and Pérez, 2022).

The objectives of this study are, on the one hand, to analyze the relationship between group cohesion, motor self-efficacy, frustration tolerance and social skills in the context of PE; and on the other hand, to examine how these variables are influenced by factors such as gender, educational stage, use of mobile devices and PA practice. The study focuses on students in primary and secondary education, with the aim of providing evidence to help understand how these factors interact in the socioemotional and motor development of students. The main hypothesis is that higher levels of group cohesion, motor self-efficacy, social skills and frustration tolerance will be related to greater PA practice, compared to those who do not, and that excessive use of mobile devices could be negatively associated with some of them, especially among girls. These findings could serve as a basis for future educational interventions that seek to promote more positive, inclusive and healthy learning environments in the field of PE.

## 2 Materials and methods

### 2.1 Participants

This study involved 807 FE students (*N* = 807) selected through non-probabilistic purposive sampling (Salkind, 1999). Among them, there were 393 boys (48.7%) and 414 girls (51.3%). The mean age of the participants was 13.06 years with a standard deviation of 1.81.

In terms of level of education, 33.3% (269) of the students belonged to Primary Education, while the remaining 66.7% (538) belonged to Secondary Education. In terms of type of educational institution, 48.3% of the students (390) attended centers in rural areas, while the remaining 51.7% (417) attended schools in urban areas.

Regarding sports practice, 68.4% (552) of the students engaged in some type of sports practice, while the remaining 31.6% (255) did not engage in any type of activity. Regarding the use of social networks, 78.2% (628 people) of the respondents claimed to use platforms such as Instagram, TikTok or Facebook, while 21.8% (175 people) do not.

The inclusion criteria were as follows: (a) informed consent of the parents or guardians, and (b) enrolled in public institutions in the autonomous community of Extremadura, at the primary or secondary education level, aged between 6 and 18 years.

Table 1 presents the sociodemographic characterization of the sample.

**Table 1:** Profile and characterization of the sample (*N* = 807).

**Variable**	**Categories**	***N***	**%**
Sex	Boy	393	48.7
	Girl	414	51.3
Course	5°Primary	96	11.9
	6° Primary	173	21.4
	1° E.S.O.	134	16.6
	2° E.S.O.	209	25.9
	3° E.S.O.	73	9.0
	4° E.S.O.	66	8.2
	Primero de bachillerato	56	6.9
Educational stage	Primary education	269	33.3
	Secundary education	538	66.7
Location of the center	Rural	390	48.3
	Urban	417	51.7
Play sports	Yes	552	68.4
	No	255	31.6
Do you have and use social media (Instagram, TikTok, or Facebook)?	Yes	628	78.2
	No	175	21.8
**Variable**		***M***	**SD**
Age		13.06	1.81
Weight		50.56	11.55
Height		1.59	0.11
Hours of daily physical activity or sports		2.25	1.303
Hours spent using mobile devices such as cell phones or tablets		2.91	1.484

*N*, number; %, percentage; SD, standard deviation; M, mean.

### 2.2 Procedures

Based on the official list of public schools in Extremadura, provided by the Consejería de Educación y Empleo de la Junta de Extremadura, contact data were collected for those schools that offered Primary Education or Compulsory Secondary Education, corresponding to ages between 6 and 18 years.

An e-mail was sent to all the identified centers, specifically addressed to the PE teachers, explaining the purpose of the research, attaching a model of the instrument to be used and including the informed consent form for families. If they were willing to participate, teachers were asked to make an appointment via e-mail so that a member of the research team could travel to the center and apply the questionnaires to the PE students, provided that all the consent forms signed by the parents or legal guardians had been previously submitted.

On the agreed date, a member of the research team went to the center and, after verifying that all the students had the required authorization, provided each participant with a tablet with access to the digital questionnaire, hosted on the Google Forms platform. Previously, physical measurements of weight and height were taken using a Tanita stadiometer and a bioimpedance meter of the same brand. These data were given to each student to be included in the corresponding questions of the questionnaire. During the administration of the instrument, the researcher read aloud each item in order to ensure correct understanding by all participants. The digitization of the questionnaire responded to the intention of streamlining both the collection and storage of the information, allowing efficient management of the data in a single database.

The average time required to complete the questionnaire was ~10 min. Data collection was conducted anonymously between February and May 2024.

The study was conducted in accordance with the guidelines established in the Declaration of Helsinki and was approved by the Ethics Committee of the EDUCA platform for excellence in educational research (approval code: 23/2025).

### 2.3 Instruments

Sociodemographic data: A seven-question questionnaire was created to determine the identity of the sample: sex, age, grade, educational stage, location of the center, sports practice and use of social networks.

Biometric measurements (height and weight): To determine the height of the participants, a Tanita Tantois stadiometer (Tanita Corporation, Tokyo, Japan) was used, positioned at a vertical angle that ensured that the scale was in a straight line with the ground. The students stood with their shoulders in a straight line and arms outstretched at their sides, and height was determined in centimeters to the nearest millimeter. Body weight measurement was performed using a bioimpedance apparatus (Tanita MC-780 MA, Tanita Corporation, Tokyo, Japan) set to “standard mode,” in which the participant's age, gender and height data were provided.

Behaviors related to physical activity and use of technology: Participants self-reported hours of PA, indicating the average daily time spent performing PA. This measure facilitated the identification of the frequency and duration with which people integrate exercise into their daily lives. To calculate the use of mobile devices, participants were asked to be aware of the average weekly time spent on the digital health application available for iOS and Android devices. This action made it possible to obtain objective and accurate information, about the time spent on cell phone use during the week.

Frustration tolerance: To assess frustration tolerance in the context of PE classes, the Stress Management Subscale belonging to the Emotional Quotient Inventory Youth Version (EQ-i: YV), created by Bar-On (2000) and later translated into Spanish by Oliva Delgado et al. (2011), was used. This instrument makes it possible to assess the vision that children and adolescents have about their ability to handle stress, manage difficult circumstances and manage their impulses. The version used in this study includes 8 items, which were evaluated through a five-point Likert scale, ranging from 1 (Never) to 5 (Always). Prior to the study of the data, the ratings of all the items were inverted to ensure a correct interpretation of the results (i.e., 1 became 5, 2 became 4, 3 remained the same, 4 became 2 and 5 became 1). Some examples of the items covered include: “I find it complicated to manage my irritation” and “When I get upset, I react without reflecting.” The Spanish-translated version was subjected to a confirmatory factor analysis, using the principal axis extraction procedure with direct oblimin rotation, in order to verify construct validity. Regarding reliability, the creators of the adaptation reported an adequate internal consistency, reaching a Cronbach's alpha coefficient of 0.77.

Social Skills Scale: The Social Skills Scale developed by Oliva Delgado et al. (2011) was used to assess the vision that young people have of their own social competencies in the educational context. This tool consists of 12 items structured in three dimensions: communicative or relational skills (items 1, 3, 5, 6, and 8), assertiveness (items 2, 7, and 11) and conflict resolution (items 4, 9, 10, and 12). Participants gave their answers on a 7-point Likert scale, ranging from 1 (absolutely false) to 7 (absolutely true), depending on the level of connection with each statement. An illustrative case is: “It is complicated to start a dialogue with someone I don't know.” It is important to note that the items with negative wording in the communicative dimension were earmarked for analysis. Regarding its psychometric characteristics, the scale showed adequate internal consistency, with a Cronbach's alpha of 0.69 in the total, and coefficients of 0.74, 0.75, and 0.80 in the dimensions of communicative and relational skills, assertiveness and conflict resolution, respectively.

Motor self-efficacy: To assess the degree of motor self-efficacy in the school group, the method validated in Spanish by Hernández Álvarez et al. (2011) was used. This questionnaire consists of 10 items that illustrate common situations in the field of physical-sports activity, and is answered through a 4-point Likert scale, where 1 equals “totally disagree” and 4 equals “totally agree.” The total score is achieved by adding the responses of the 10 items, with a scale ranging from 10 to 40 points. Lower values indicate low motor self-efficacy, while higher values indicate a high perception of competence in the motor domain. In terms of reliability, the instrument showed adequate internal consistency, with a Cronbach's alpha coefficient of 0.82.

Group cohesion in Physical Education: To measure group unity within the framework of PE, the Group Cohesion Evaluation Questionnaire (GCEQ) was used, initially created by Glass and Benshoff (2002) and later translated and validated in Spanish by Rojo-Ramos et al. (2024). This instrument includes nine items that address different facets of cohesion, such as the feeling of belonging, collaboration among members and reciprocal support. The statements are answered through a 4-point Likert scale, ranging from 1 (“Not at all similar to me/my group”) to 4 (“Exactly like me/my group”). They include some examples of items: “We manage well together” and “We motivate each other in challenges.” In terms of reliability, the questionnaire showed excellent levels of internal consistency, with a Cronbach's alpha of 0.927 and a McDonald Omega coefficient of 0.928.

### 2.4 Statistical analysis

First, the distribution of the data was examined in order to confirm compliance with the assumed normality, a necessary condition for choosing the appropriate statistical tests. For this purpose, the Kolmogorov-Smirnov test was used, and the results showed that the data presented an abnormal distribution (*p* < 0.005). In view of this circumstance, it was decided to use non-parametric statistical tests.

The Mann–Whitney test was used to examine the differences in the scores of the variables group cohesion, frustration tolerance, social skills and motor efficiency in relation to categorical variables such as sex, geographic location, use of social media, and club sports practice. A statistical significance level of *p* < 0.05 was established in all analyses.

Additionally, Spearman's Rho correlation coefficient was used to examine the relationship between the dimensions mentioned and the sociodemographic variables. Mondragón Barrera (2014) proposed the following criteria to interpret the strength of the correlations: values between 0.01 and 0.10 are interpreted as low correlation, values between 0.11 and 0.50 as moderate correlation, values between 0.51 and 0.75 as strong correlation, values between 0.76 and 0.90 as very strong correlation, and values above 0.91 as perfect correlation.

The Hedges' g statistic was used to determine the effect size of the comparisons made according to sex, demographic location, use of social networks and sports practice. Cohen (2013) criteria establish that an effect is absent if the value is < 0.20, small if the value is between 0.21 and 0.49, moderate if the value is between 0.50 and 0.79, and large if the value is >0.80.

To better understand the relationship between the variables analyzed and to predict group cohesion, frustration tolerance, social skills and motor efficiency scores, a multiple regression analysis was performed using the stepwise selection method. In this process, variables were added to the predictive models whenever they met the statistical significance threshold of *p* < 0.05.

The reliability of the instruments used was assessed using Cronbach's alpha and McDonald's omega coefficients, which were calculated for each of the dimensions measured. According to the parameters established by Nunnally (1994), values below 0.70 are considered indicators of low reliability, values between 0.71 and 0.90 indicate acceptable reliability, and values above 0.91 indicate excellent reliability. The Omega coefficient was calculated using the maximum likelihood model (Omega ML).

Finally, the sociodemographic data were presented using absolute frequencies (N) and percentages, while the points of the study variables were described using measures of central tendency and dispersion, specifically the mean (M) and standard deviation (SD). The Statistical Package for the Social Sciences (SPSS), version 23 for Mac, was used to perform all statistical analyses.

## 3 Results

Mean scores (M), standard deviations (SD), significant differences (p) and Hedges effect sizes (g) for group cohesion, motor self-efficacy, frustration tolerance and social skills, based on gender, demographic location, social network use and sport practice are shown in Table 2. Significant differences in favor of females were found for assertiveness (*p* < 0.001, g = 0.17), frustration tolerance (*p* = 0.014, g = 0.23), communication and relational skills (*p* = 0.001, g = 0.16), and conflict resolution skills (*p* = 0.026, g = 0.43). Although girls also scored higher on group cohesion and motor self-efficacy, these differences were only slight (g = 0.29 and g = 0.13, respectively). In the case of self-efficacy they did not reach statistical significance (*p* = 0.065). In terms of demographic location, participants from rural areas had significantly higher scores on motor self-efficacy (p = 0.003, g = 0.21), communication and relational skills (p < 0.001, g = 0.19), conflict resolution skills (*p* = 0.007, g = 0.22), and assertiveness (*p* = 0.001, g = 0.4). In addition, compared to those in urban areas, they also showed higher frustration tolerance (*p* < 0.001, g = 0.36). There were significant differences in group cohesion (*p* < 0.001, g = 0.37), communication skills (*p* < 0.001, g = 0.23), and conflict resolution skills (*p* = 0.006, g = 0.02) in relation to social network use, with the highest percentages of respondents reporting not using these platforms. However, effect sizes were generally small. There were no discernible differences in motor self-efficacy, frustration tolerance, or assertiveness in the use of social networks. Finally, with respect to sports practice, statistically significant differences were found in motor self-efficacy (*p* = 0.016, g = 0.18), favoring those who practice sports regularly. In addition, there were slightly higher scores in group cohesion (*p* = 0.142, g = 0.11) and conflict resolution skills (*p* = 0.226, g = 0.11), although they were not statistically significant. Differences in assertiveness, frustration tolerance and communication skills were also smaller.

**Table 2:** Scores, differences, and effect sizes of resilience, self-esteem, and fitness by sex, demographic location, and social media use.

**Construct**	**Dimension**	**Sex**				**Ubication**				**Use of R.R.S.S**.				**Sports practices**			
		**Male**	**Female**			**Rural**	**Urban**			**Yes**	**No**			**Yes**	**No**		
		**M (SD)**	**M (SD)**	***p***	***g***	**M (SD)**	**M (SD)**	***p***	***g***	**M (SD)**	**M (SD)**	***p***	***g***	**M (SD)**	**M (SD)**	***p***	***g***
Group cohesion, motor self-efficacy		3.355 (0.50), 30.35 (3.49)	3.503 (0.43), 31.89 (4.58)	< 0.001 0.065^*^	0.29 0.13	3.43 (0.42), 30.15 (3.80)	3.42 (0.51), 32.11 (4.21)	0.890 0.003	0.01 0.21	3.26 (0.57), 29.80 (4.13)	3.47 (0.43), 31.50 (3.90)	< 0.001 0.329^*^	0.37 0.08	3.44 (0.47), 31.89 (4.27)	3.39 (0.46), 29.52 (3.70)	0.142 0.016	0.11 0.18
Frustration tolerance		2.371 (0.80)	2.51 (0.75)	0.014	0.23	2.58 (0.79)	2.31 (0.74)	< 0.001	0.36	2.52 (0.82)	2.42 (0.76)	0.962^*^	0.34	2.46 (0.75)	2.41 (0.84)	0.423^*^	0.05
Social skills	Communication and relational skills	3.717 (1.52)	3.92 (1.39)	0.001	0.16	4.11 (1.45)	3.55 (1.40)	< 0.001	0.19	4.37 (1.23)	3.68 (1.48)	< 0.001	0.23	3.85 (1.34)	3.76 (1.68)	0.525^*^	0.09
	Conflict resolution skills	5.221 (1.26)	5.45 (1.27)	0.026	0.43	5.43 (1.32)	5.24 (1.21)	0.007	0.22	5.11 (1.36)	5.40 (1.23)	0.006	0.02	5.35 (1.26)	5.29 (1.30)	0.226^*^	0.11
	Assertiveness	4.430 (1.38)	4.88 (1.13)	< 0.001	0.17	4.80 (1.21)	4.54 (1.32)	0.001	0.4	4.56 (1.45)	4.70 (1.22)	0.791^*^	0.01	4.61 (1.33)	4.79 (1.12)	0.157^*^	0.06

Valor de *p* es significativo < 0.05^*^. M, valor medio; SD, desviación estándar.

Table 3 shows the correlation between age, PA hours and time spent using mobile devices, in addition to the dimensions of group cohesion, motor self-efficacy, frustration tolerance and social skills. The findings indicate that group cohesion presents a moderately positive correlation with age in the whole sample (ρ = 0.13, *p* < 0.001) and in girls (ρ = 0.14, *p* = 0.00), despite the fact that this correlation is slightly less pronounced in boys (ρ = 0.11, *p* = 0.029). This indicates that, as individuals get older, they tend to notice a slight increase in group unity. Similarly, group cohesion and hours of PA also show a moderately high correlation in the total sample (ρ = 0.16, *p* < 0.001), with a stronger correlation in boys (ρ = 0.21, *p* < 0.001) than in girls (ρ = 0.14, *p* = 0.00), indicating that those who perform more physical exercise experience greater cohesion in their group. In addition, a moderately positive correlation was detected between group cohesion and the time allocated to the general use of mobile devices (ρ = 0.17, *p* < 0.001), this correlation being slightly higher among girls (ρ = 0.14, *p* = 0.00). This suggests that those who use these devices more tend to feel more connected to their social group.

**Table 3:** Correlation between the variables group cohesion, motor self-efficacy, frustration tolerance and social skills and the variables age, hours of physical activity and daily hours of mobile device use.

**Construct**	**Dimension**	**Age** ρ ***(p)***			**Hours of physical activity** *ρ **(p)***			**Hours of mobile device use** *ρ **(p)***		
		**Total**	**Male**	**Female**	**Total**	**Male**	**Female**	**Total**	**Male**	**Female**
Group cohesion		0.13 (< 0.001)	0.11 (0.029)	0.14 (0.00)	0.16 (< 0.001)	0.21 (< 0.001)	0.14 (0.00)	0.17 (< 0.001)	0.14 (0.00)	0.17 (< 0.001)
Motor self-efficacy		0.04 (0.21)	0.17 (0.00)	−0.08 (0.10)	0.28 (< 0.001)	0.27 (< 0.001)	0.26 (< 0.001)	−0.08 (0.82)	0.05 (0.29)	−0.06 (0.22)
Frustration tolerance		−0.17 (< 0.001)	−0.13 (0.01)	−0.25 (< 0.001)	−0.03 (0.38)	−0.07 (0.16)	0.07 (0.16)	−0.03 (0.36)	−0.08 (0.11)	0.01 (0.85)
Social skills	Communication and relational skills	−0.39 (< 0.001)	−0.36 (< 0.001)	−0.44 (< 0.001)	−0.06 (0.08)	−0.01 (0.03)	0.01 (0.75)	−0.19 (< 0.001)	−0.29 (< 0.001)	−0.12 (0.01)
	Conflict resolution skills	−0.11 (0.00)	−0.08 (0.10)	−0.14 (0.00)	0.08 (0.01)	0.02 (0.62)	0.17 (< 0.001)	−0.08 (0.02)	0.10 (0.04)	0.04 (0.34)
	Assertiveness	0.07 (0.04)	0.11 (0.02)	0.04 (0.32)	−0.08 (0.01)	−0.00 (0.95)	−0.13 (0.00)	0.03 (0.33)	−0.05 (0.32)	0.10 (0.04)

Regarding motor self-efficacy, age did not show a significant correlation in the total sample (ρ = 0.04, *p* = 0.21). However, in boys, a moderately positive correlation was observed (ρ = 0.17, *p* = 0.00), while in girls, the correlation was negative, although small (ρ = −0.08, *p* = 0.10). This could indicate that boys tend to improve their motor self-efficacy as they grow older, while girls seem to experience the opposite. On the other hand, motor self-efficacy did show a moderately positive correlation with PA hours (ρ = 0.28, *p* < 0.001) in the total sample, with similar results in boys (ρ = 0.27, *p* < 0.001) and girls (ρ = 0.26, *p* < 0.001). This supports the idea that practicing PA is related to greater confidence in motor skills. Regarding time spent using mobile devices, a very weak negative correlation was found in the general population (ρ = −0.08; *p* = 0.82), a weak positive correlation in boys (ρ = 0.05; *p* = 0.29), and a slight negative correlation in girls (ρ = −0.06; *p* = 0.22). This suggests no significant relationship between mobile device use and perceived motor self-efficacy.

Regarding frustration tolerance, age showed a moderately negative correlation in the total sample (ρ = −0.17, *p* < 0.001), being more pronounced in girls (ρ = −0.25, *p* < 0.001) than in boys (ρ = −0.13, *p* = 0.01). This indicates that frustration tolerance tends to decrease over time, particularly in girls. No significant links were found between PA hours and frustration tolerance, nor between hours spent using mobile devices, suggesting that these factors do not appear to have a significant impact on how people perceive their ability to handle frustrating situations.

Concerning communication and relational skills, a moderately negative correlation with age was observed in the analyzed sample (ρ = −0.39, *p* < 0.001), both in boys (ρ = −0.36, *p* < 0.001) and girls (ρ = −0.44, *p* < 0.001). This indicates that, as people get older, they tend to view these skills less positively, with girls showing greater sensitivity to this change. Regarding hours dedicated to PA, the correlation was weak and not significant overall (ρ =-0.06, *p* = 0.08), very weak in girls (ρ = −0.01, *p* = 0.03), and slightly positive but not significant in boys (ρ = 0.01, *p* = 0.75). When analyzing the time spent using mobile devices, a significant negative correlation was found in girls (ρ = −0.29; *p* < 0.001) and in the general population (ρ = −0.19; *p* < 0.001), while the correlation was low and not significant in boys (ρ = −0.12; *p* = 0.01). This suggests that greater use of these devices is linked to a lower perception of communication skills, especially among girls.

In relation to conflict resolution skills, a slight negative correlation with age was observed in the general sample (ρ = −0.11, *p* = 0.00) and in girls (ρ = −0.14, *p* = 0.00). On the other hand, hours spent engaged in PA showed a weak but significant positive correlation in the general population (ρ = 0.08, *p* = 0.01) and in girls (ρ = 0.17, *p* < 0.001). However, no significant correlation was found in boys (ρ = 0.02, *p* = 0.62). This may indicate that engaging in PA may be associated with improved conflict resolution skills, especially among girls. In contrast, time spent using mobile devices showed a weak but significant negative correlation in the total sample (ρ = −0.08, *p* = 0.02), indicating that greater use of mobile devices may be associated with lower conflict resolution skills. However, a weak and significant positive correlation was found in boys (ρ = 0.10, *p* = 0.04), while in girls there was no significant correlation (ρ = 0.04, *p* = 0.34), suggesting that boys may have a greater capacity to resolve conflicts compared to girls.

In summary, a slight positive correlation was observed between age and assertiveness in the overall sample (ρ = 0.07, *p* = 0.04) and in girls (ρ = 0.11, *p* = 0.02), although this was not found in boys (ρ = 0.04, *p* = 0.32). This suggests that students tend to become more self-confident as they grow older. Regarding PA hours, a negative correlation was found in the total (ρ = −0.08, *p* = 0.01), with no effect in boys (ρ = −0.00, *p* = 0.95), and a negative correlation in girls (ρ = −0.13, *p* = 0.00). This indicates that, especially in girls, greater PA practice could be related to lower perceptions of assertiveness. Regarding mobile device use, a positive, though not significant, correlation was noted in the overall sample (ρ = 0.03, *p* = 0.33), a negative, but not significant, correlation was noted in boys (ρ = −0.05, *p* = 0.32), and a positive, but significant, correlation was noted in girls (ρ = 0.10, *p* = 0.04). This could suggest that, for girls, greater use of these devices is linked to greater perceptions of assertiveness.

Table 4 shows the predictive models obtained through linear regression for the studied variables (group cohesion, motor self-efficacy, frustration tolerance, and social skills). In the regression model for group cohesion (*R*^2^ = 0.36), several significant predictors were identified: motor self-efficacy (β = 0.04, *p* < 0.001), use of mobile devices (β = 0.06, *p* < 0.001), sex (β = 0.17, *p* < 0.001), hours of PA (β = 0.04, *p* < 0.001), location (β = 0.10, *p* < 0.001), and frustration tolerance (β = 0.08, *p* < 0.001). These findings suggest that both personal, behavioral, and contextual factors play an important role in how group cohesion is perceived. On the other hand, communication and relational skills showed a significant negative relationship with cohesion (β = −0.04, *p* < 0.001), which could indicate an inverse effect between social interaction and the sense of belonging to a group. Furthermore, assertiveness (β = 0.03, *p* = 0.00) and sports practice (β = 0.06, *p* = 0.03) also contributed positively to the model.

**Table 4:** Predictive models for group cohesion, motor self-efficacy, frustration tolerance, and social skills.

**Variable**	**β**	**SE**	***t***	***p***
**Group cohesion: model 1 (R2)** = **0.36**				
Motor self-efficacy	0.04	0.00	11.25	(< 0.001)
Screen time	0.06	0.01	6.08	(< 0.001)
Sex	0.17	0.03	5.75	(< 0.001)
Physical activity hours	0.04	0.01	4.16	(< 0.001)
HHSS conflict resolution	0.02	0.01	1.78	0.07
Location	0.10	0.03	3.39	(< 0.001)
Frustration tolerance	0.08	0.02	4.14	(< 0.001)
HHSS communication and relationship	−0.04	0.01	−3.67	(< 0.001)
HHSS assertiveness	0.03	0.01	2.61	0.00
Sport practice	0.06	0.03	2.06	0.03
Constant	1.21	0.15	8.08	(< 0.001)
**Motor self-efficacy: model 1 (R2)** = **0.02**				
HHSS assertiveness	1.03	1.12	0.92	0.35
Hours of physical activity	4.07	1.08	3.77	(< 0.001)
HHSS communication and relationship	0.01	0.95	0.01	0.98
Sex	2.90	2.83	1.02	0.30
Location	1.48	2.83	0.52	0.60
Screen time	1.37	0.94	−1.44	0.14
Constant	18.85	7.87	2.39	0.01
**Frustration tolerance: model 1 (R2)** = **0.17**				
HHSS communication and relationship	0.19	0.01	10.16	(< 0.001)
Location	−0.21	0.05	−3.99	(< 0.001)
HHSS assertiveness	−0.06	0.02	−2.93	0.00
Hours of physical activity	−0.03	0.02	−1.61	0.10
Constant	2.17	0.13	15.73	(< 0.001)
**HHSS, communicative and relational: model 1 (R2)** = **0.26**				
Frustration tolerance	0.61	0.06	8.98	(< 0.001)
Height	−2.45	0.45	−5.45	(< 0.001)
Group cohesion	−0.51	0.12	−4.06	(< 0.001)
HHSS assetiveness	0.21	0.04	4.91	(< 0.001)
Hours of physical activity	0.13	0.04	3.19	0.00
Motor self-efficacy	−0.04	0.16	−3.14	0.00
Location	−0.35	0.10	−3.35	(< 0.001)
Constant	8.39	0.86	9.70	(< 0.001)
**HHSS, assertiveness: model 1 (R2)** = **0.44**				
HHSS conflict resolution	0.51	0.03	16.18	(< 0.001)
Motor self-efficacy	0.07	0.01	6.65	(< 0.001)
Height	1.85	0.34	5.40	(< 0.001)
Sex	0.32	0.07	4.09	(< 0.001)
HHSS communication and relationship	0.12	0.02	4.24	(< 0.001)
Group cohesion	0.29	0.09	3.04	0.00
Hours of physical activity	−0.09	0.03	−3.01	0.00
Frustration tolerance	−0.14	0.05	−2.61	0.00
Constant	−4.35	0.70	−6.18	(< 0.001)
**HHSS, conflict resolution: model 1 (R2)** = **0.36**				
HHSS assertiveness	0.54	0.03	17.95	(< 0.001)
Height	−2.51	0.57	−4.35	(< 0.001)
Weight	0.01	0.00	2.00	0.04
Constant	6.21	0.70	8.84	(< 0.001)

Regarding motor self-efficacy (*R*^2^ = 0.02), the only significant predictor variable was time spent engaged in PA (β = 4.07, *p* < 0.001), suggesting that greater PA practice may be related to greater perceptions of motor self-efficacy. The other variables included in the model did not show significant relationships with motor self-efficacy.

In the regression analysis on frustration tolerance (*R*^2^ = 0.17), the most significant predictor variable was communication and relational skills (β = 0.19, *p* < 0.001). This suggests that having better communication and relational skills may be related to a greater ability to handle frustrating situations. Furthermore, significant negative relationships were identified with location (β = −0.21, *p* < 0.001) and assertiveness (β = −0.06, *p* = 0.00), indicating that certain environments or a more direct communication style could be linked to lower frustration tolerance. On the other hand, PA hours also showed a negative but non-significant relationship (β = −0.03, *p* = 0.10).

In the regression model for communication and relational skills (*R*^2^ = 0.26), the most influential variable was frustration tolerance (β = 0.61, *p* < 0.001), implying that a greater ability to tolerate frustrating situations is associated with a higher perception of communication skills. Positive and significant effects were also found for assertiveness (β = 0.21, *p* < 0.001) and PA hours (β = 0.13, *p* = 0.00), suggesting that both the ability to express oneself directly and regular PA practice benefit these types of skills. In contrast, several variables showed negative relationships with this dimension: height (β = −2.45, *p* < 0.001), group cohesion (β = −0.51, *p* < 0.001), motor self-efficacy (β = −0.04, *p* = 0.00) and location (β = −0.35, *p* < 0.001).

In the regression analysis on assertiveness (*R*^2^ = 0.44), conflict resolution was found to be the most influential predictor (β = 0.51, *p* < 0.001). This suggests that having a greater ability to resolve conflicts is strongly related to an increase in assertiveness. In addition, other factors such as motor self-efficacy (β = 0.07, *p* < 0.001), height (β = 1.85, *p* < 0.001), sex (β = 0.32, *p* < 0.001), communication and relational skills (β = 0.12, *p* < 0.001), and group cohesion (β = 0.29, *p* = 0.00), showing positive and statistically significant effects. On the other hand, negative relationships were identified with PA hours (β = −0.09, *p* = 0.00) and frustration tolerance (β = −0.14, *p* = 0.00), suggesting that more time spent on PA or greater frustration sensitivity could be linked to lower levels of assertiveness.

In the regression model for conflict resolution (*R*^2^ = 0.36), assertiveness was the most significant predictor (β = 0.54, *p* < 0.001), indicating that a better ability to express oneself appropriately is directly related to greater conflict resolution skills. A significant negative effect of height was also observed (β = −2.51, *p* < 0.001), suggesting that physical factors such as height may be inversely related to this social competency. Furthermore, weight showed a positive relationship (β = 0.01, *p* = 0.04), although with a low impact.

Finally, Table 5 presents the reliability coefficients of the scales used in the study. All the dimensions evaluated showed acceptable levels of internal consistency, with Cronbach's alpha and McDonald's omega values within satisfactory ranges. In particular, group cohesion obtained the highest coefficients (α = 0.846, ω = 0.847), followed by motor self-efficacy (α = 0.812, ω = 0.808) and frustration tolerance (α = 0.788, ω = 0.792), indicating high reliability in the measurement of these variables. Likewise, social skills in their communicative and relational dimensions presented adequate values (α = 0.741, ω = 0.739). On the other hand, the dimensions of conflict resolution (α = 0.523, ω = 0.526) and assertiveness (α = 0.681, ω = 0.680) showed lower coefficients, although within a range considered acceptable in exploratory contexts. Overall, the results support adequate reliability of the scales used, in accordance with the criteria established by Nunnally (1994).

**Table 5:** Internal consistency coefficients of each variable studied.

**Variables**	**Cronbach's alpha**	**McDonald's omega**
Group cohesion	0.846	0.847
Motor self-efficacy	0.812	0.808
HHSS commuunicative and relational	0.741	0.739
HHSS conflict resolution	0.523	0.526
HHSS assertiveness	0.681	0.680
Frustration tolerance	0.788	0.792

## 4 Discussion

The present study aimed to analyze the relationship between group cohesion, motor self-efficacy, frustration tolerance, social skills, and various sociodemographic variables, such as age, sex, geographic location, PA practice, and use of electronic devices, in primary and secondary school students. To this end, a detailed analysis of the results obtained for each main variable was conducted in relation to the proposed hypotheses, which proposed a positive association between higher levels of group cohesion, motor self-efficacy, frustration tolerance, and social skills with greater PA practice, and a negative relationship with excessive use of mobile devices, especially among girls. The findings show differentiated patterns based on sex, sports practice, and use of electronic devices, highlighting the positive influence of PA practice on variables such as group cohesion and motor self-efficacy, and a negative relationship between excessive use of mobile devices and communication and relational skills. These results may contribute to a greater understanding of how social, technological, and demographic factors interact with adolescents' personal and social development.

Regarding group cohesion, the results of this study indicate that, as age increases, the perception of cohesion also tends to increase slightly, especially in girls. This pattern may be related to greater social maturity and to the fact that, over time, students adopt more collaborative and supportive attitudes within the group, as pointed out by Guillén and Sandoval (2022) and Martínez et al. (2021). Likewise, a positive correlation was found between group cohesion and hours of PA, stronger in boys, suggesting that greater involvement in physical and sports activities is associated with a stronger perception of group unity. These results coincide with those reported by Carron et al. (1996), who found that more cohesive groups showed higher levels of adherence to exercise. Furthermore, a positive average correlation was observed between group cohesion and the use of mobile devices, which could be explained by the role that these devices play as socialization tools among adolescents; in this sense, Vidales-Bolaños and Sádaba (2017) highlight that moderate use and social interaction-oriented use can strengthen group ties. On the other hand, it is also relevant to consider gender differences in the way in which PA is practiced, since girls tend to prefer individual activities due to their orientation toward physical wellbeing rather than competition (Castro Sánchez et al., 2016; Martínez-Benítez et al., 2017), which could be influenced by a more competitive environment in male groups (Lavega Burgués et al., 2012), thus differentially affecting the perception of group cohesion.

Regarding motor self-efficacy, age did not show a significant relationship, although it could be suggested that boys perceive an improvement in their motor self-efficacy with age, while the opposite is true for girls. According to several studies, the arrival of adolescence in both boys and girls is accompanied by a progressive decline in self-efficacy (Carrasco Ortiz and Del Barrio Gándara, 2002; Hernández Álvarez et al., 2008), so this does not coincide with the results obtained in this study. This decline in motor self-efficacy with the arrival of adolescence may be related to the abandonment of PA that occurs after the age of 12, especially among girls (Hernández Álvarez et al., 2008). On the other hand, motor self-efficacy showed a medium positive correlation with hours of PA, which reinforces the idea that practicing PA is related to greater confidence in one's motor skills. Several studies (Fraile García et al., 2019; Muñoz and García, 2013) concur with the results obtained in this study, finding a significant relationship between motor self-efficacy and PA level. Regarding the hours of mobile device use, it is observed that the use of electronic devices does not have a significant relationship with the perception of motor self-efficacy. In this sense, it is worth mentioning that there are still few publications that observe the relationship between motor self-efficacy and the use of mobile devices, but there are studies that study the relationship between PA practice, mobile use, and self-concept. The systematic review carried out by Zagalaz-Sánchez et al. (2019) compiled research that shows both favorable and unfavorable associations between PA and mobile device use; however, they state that young people who regularly practice PA spend less time on the Internet than those who do not (Ferreiro et al., 2017). It is also important to note that mobile devices can be used beneficially, as there are technological applications that promote healthy lifestyle habits (Zach et al., 2016). In fact, several authors have indicated that the use of these applications may be associated with greater motivation to regularly practice PA during leisure time (Zach et al., 2016).

Regarding frustration tolerance, age showed a more marked relationship in girls than in boys, suggesting that, as age increases, frustration tolerance tends to decrease, especially in girls. In this sense, Sorrenti et al. (2015) evaluated the relationship between age and frustration intolerance in university students and found that older university students tend to tolerate frustration better than younger ones, these results coinciding with those obtained in this study. On the other hand, there is the publication by Ibañez et al. (2018), who found no significant differences between both groups (one group of 14–16 years old and another of 17–19 years old) with respect to frustration tolerance. Finally, it is worth mentioning the publication by Medrano et al. (2018), who obtained a positive correlation between age and frustration tolerance, that is, the older the age, the greater the frustration tolerance in a group of young adults (18–30 years old). These publications (Ibañez et al., 2018; Medrano et al., 2018; Sorrenti et al., 2015) emphasize the lack of specific background on the relationship between age and frustration tolerance, therefore, the topic is novel and relevant, not only to understand this association, but also because of how the results may differ depending on the population analyzed.

On the other hand, in the area of social skills, it was observed that communication and relational skills have a moderate negative correlation with age in the total sample. This suggests that, as people grow older, these skills tend to decline, this being more noticeable in girls. A study published by Sierra-Sánchez et al. (2021) found a greater presence of shy behaviors in females, suggesting that shyness can negatively affect social skills in women as they age. Peñalva-Vélez et al. (2020) found higher levels of difficulties in social skills, especially among females from the age of 10. Regarding the time spent using mobile devices, a moderate negative correlation was observed with social skills. This suggests that, as the time spent using electronic devices increases, there may be a decrease in the ability to communicate and relate to others. The publication by Twenge and Campbell (2018) found that more daily hours of screen use were associated with lower psychological wellbeing, including lower self-control, greater difficulty making friends, and lower emotional stability, coinciding with the results obtained in this study. Other publications (Paulich et al., 2021; Yu et al., 2025) also coincided with the results of this study, finding a negative association between screen time and communication and relational skills. Excessive use of mobile devices can limit opportunities for face-to-face interaction, which are essential for the development of social skills; this could explain its negative association with communication and interpersonal relationships (Paulich et al., 2021; Twenge and Campbell, 2018; Yu et al., 2025).

Regarding conflict resolution skills, age was observed to have a low negative correlation in the total sample and in girls. This indicates that, for girls, as they grow older, there seems to be a slight tendency toward lesser conflict management skills. According to research by Lindeman et al. (1997), girls tend to resort to withdrawal strategies more frequently than boys, suggesting that, as they grow older, they may be declining in their use of effective conflict resolution methods. On the other hand, Van Doorn et al. (2011) found that, in some cases, conflict resolution skills do not improve with age in girls. These findings could be related to several factors, including interpersonal conflicts during adolescence, increasing social pressure, and the emotional and hormonal changes that are part of female development (Bámaca-Colbert et al., 2012). Furthermore, a low positive correlation was noted between these skills and PA hours in the total sample, suggesting that PA could help improve conflict resolution, especially in girls. As Fernández-Rio and Méndez-Giménez (2016) mention, traditional methodologies often minimize the learning of socio-affective skills. In contrast, methodologies based on games and cooperative learning (Velázquez Callado, 2008) encourage the improvement of these skills. In this regard, we find the publication by Polvi and Telama (2000), who conclude that it is possible to encourage helping behaviors in the school environment if students are given the opportunity to practice with different peers. Cooperative learning, especially when structured to promote diverse interactions, can be an effective tool for improving social skills such as conflict resolution (Polvi and Telama, 2000). Finally, regarding the number of hours spent using mobile devices, a significant correlation was observed in the overall sample, suggesting that greater use of electronic devices could be associated with a lower ability to resolve conflicts. Research by Tandoc et al. (2015) investigates how social media use, envy, and conflict resolution skills are related among students. The findings suggest that intensive social media use could be associated with poor interpersonal skills, which in turn negatively impacts the ability to resolve conflicts, both in academic and social settings (Tandoc et al., 2015). This conclusion is consistent with the results of this study.

Finally, regarding assertiveness, age was observed to have a low positive correlation in the total sample, but not in girls. This could suggest that, as they age, boys tend to develop a higher level of assertiveness. Several studies (Bourke, 2002; Das and Shah, 2013) support these findings, indicating that boys show higher levels of assertiveness. Regarding PA hours, it was noted that more practice could be related to a lower perception of assertiveness, especially among girls. A study published by Babic et al. (2014) observed that for girls, the benefits are not always proportional to their perceived social skills. This effect may be influenced by social pressures and gender differences (Babic et al., 2014). On the other hand, mobile device hours showed a positive, albeit low, correlation with assertiveness in the total sample and in girls. This suggests that greater use of these devices could be linked to higher perceptions of assertiveness, especially among girls. Most recent studies have focused on the negative effects of excessive mobile device use on adolescents' mental health, particularly in girls, such as symptoms of depression, lower psychological wellbeing, and problems in social relationships (Paulich et al., 2021; Twenge and Campbell, 2018). Therefore, although this finding indicates a possible positive relationship between mobile device use and perceptions of assertiveness, especially among girls, further research is needed to specifically analyze this association, considering variables such as the social context in which it occurs.

### 4.1 Practical implications

This study has demonstrated the relevance of group cohesion, motor self-efficacy, frustration tolerance, and social skills within the context of physical education, and how these variables relate to age, sex, PA practice, and mobile device use. Promoting PA not only benefits the physical health of children and adolescents but also reinforces their sense of belonging to a group and improves their perception of motor competence. Similarly, applying active and cooperative methodologies in the classroom, such as team games or collaborative projects, can help students improve their social skills, especially with regard to conflict resolution and assertiveness. It is also important to pay special attention to emotional development, especially in girls, by including activities that promote emotional management from an early age, as this can have a positive impact on both the classroom environment and student wellbeing. Finally, given the constant presence of mobile devices in adolescents' lives, it would be interesting to leverage their use for educational purposes. Based on these results, it seems essential that educational programs take these factors into account, as they can significantly contribute to students' comprehensive development.

## 5 Conclusion

This research highlights the relevance of variables such as group cohesion, motor self-efficacy, frustration tolerance, and social skills in primary and secondary school students in the context of physical activity, as well as their relationship with sociodemographic factors, sports practice, and mobile device use. In this regard, positive correlations were observed between group cohesion and PA practice, especially in boys, while in girls, stronger associations were found between mobile device use and lower motor self-efficacy or communication skills. Furthermore, the results show that increasing age may be related to a decrease in frustration tolerance and social skills, especially in girls, suggesting the need to implement specific strategies according to age group and gender. Furthermore, significant differences were detected in variables such as assertiveness, self-efficacy, and cohesion based on sports practice, reinforcing the importance of PA in students' socioemotional development. Among all statistical findings, frustration tolerance was found to be the strongest predictor of communication and relational skills, underscoring the critical role of emotional regulation in the development of social competence. Based on the findings of this study, it seems relevant to further investigate how these variables influence the school context according to the educational stage, whether primary or secondary, as well as to explore the possible differences between the first and second cycles of compulsory secondary education. This would allow for the design of educational interventions more tailored to the needs of students according to their age, grade, and gender, fostering a healthier, more cohesive, and emotionally balanced environment that promotes adherence to active and healthy lifestyles.

### 5.1 Limitations and future lines of research

Regarding the study's limitations, it is worth noting that the sample is slightly unbalanced, with a greater predominance of secondary education students than primary school students. This difference in the representation of stages could have influenced the results obtained, especially in those variables that tend to change with age, such as frustration tolerance or social skills. Therefore, future research would recommend expanding the age range of the sample, including more balanced students from higher grades such as 3rd and 4th year of compulsory secondary education, as well as from high school, to observe how variables such as motor self-efficacy, group cohesion, and social skills evolve at more advanced stages of adolescent development. This would allow for a more in-depth analysis of the differences associated with maturational growth and the design of more specific interventions based on the developmental stage.

## Data Availability

The raw data supporting the conclusions of this article will be made available by the authors, without undue reservation.
